# Pleomorphic Fibroma Presenting as a Nodular Lesion in the Left Shoulder: A Case Report

**DOI:** 10.7759/cureus.37018

**Published:** 2023-04-01

**Authors:** Hatoon M Althobaiti, Ahmed Niyazi, Bader A Alharbi, Mohammed Alqahtani, Mohammad Basendwh

**Affiliations:** 1 Department of Medicine, King Abdulaziz Medical City, Jeddah, SAU; 2 Department of Medicine and Surgery, Umm Al-Qura University, Mecca, SAU; 3 Department of Dermatology, King Fahad Armed Forces Hospital, Jeddah, SAU

**Keywords:** benign skin tumor, cutaneous neoplasm, neuclear atypia, pleomorphic cells, pleomorphic fibroma

## Abstract

Pleomorphic fibroma is an uncommon benign cutaneous tumor that often presents as a single asymptomatic skin-colored lesion with indefinite clinical diagnostic features. Here, we report a case of a 47-year-old female diagnosed with pleomorphic fibroma of the skin in the left shoulder and discuss the importance of immunohistochemistry and special features in histopathology to distinguish some of the differentials.

## Introduction

Pleomorphic fibroma (PF) is a rare benign tumor that was first identified by Kamino et al. in 1989 [1]. The tumor often develops in adults and has a slight sex bias in favor of women [2]. This type of fibroma prefers the lower extremities, followed by the trunk, head, and neck, and is less frequently observed in the subungual region [3]. PF manifests as asymptomatic, solitary, skin-colored, dome-shaped, or polypoid papules that range in size from a few millimeters to almost 2 cm in diameter. Clinically, PF mimics many neoplasms including atypical fibroxanthoma (AFX), neurofibromas, and atypical lipomatous tumors. The histopathology of PF is characterized by hypocellular proliferation, nuclear pleomorphism of spindle cells, and multi-nucleated giant cells without mitosis-given that the atypia is attributed to degenerative changes rather than malignancy [2].

## Case presentation

**Figure 1:**
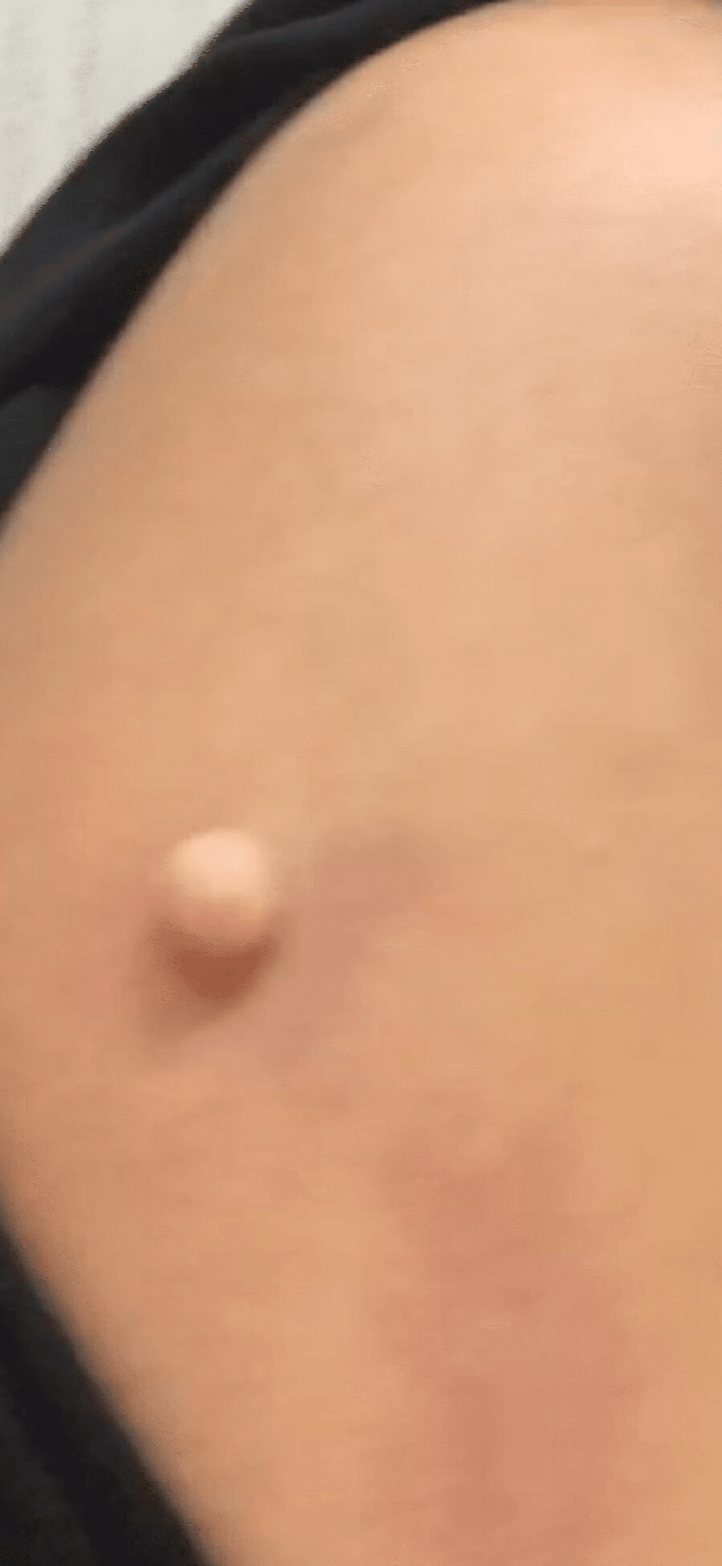
Single, round, well-demarcated 1 x 1 cm skin-colored nodule on the left arm.

A 47-year-old medically-free female presented with a history of an asymptomatic lesion on the left arm that had slowly grown over two years. The patient’s family history was unremarkable. Physical examination revealed a solitary, round, well-defined, skin-colored nodule measuring 1 x 1 cm (Figure 1). The nodule was not painful but caused slight discomfort with no discharge or bleeding. No other similar lesions were observed on the body.

**Figure 2:**
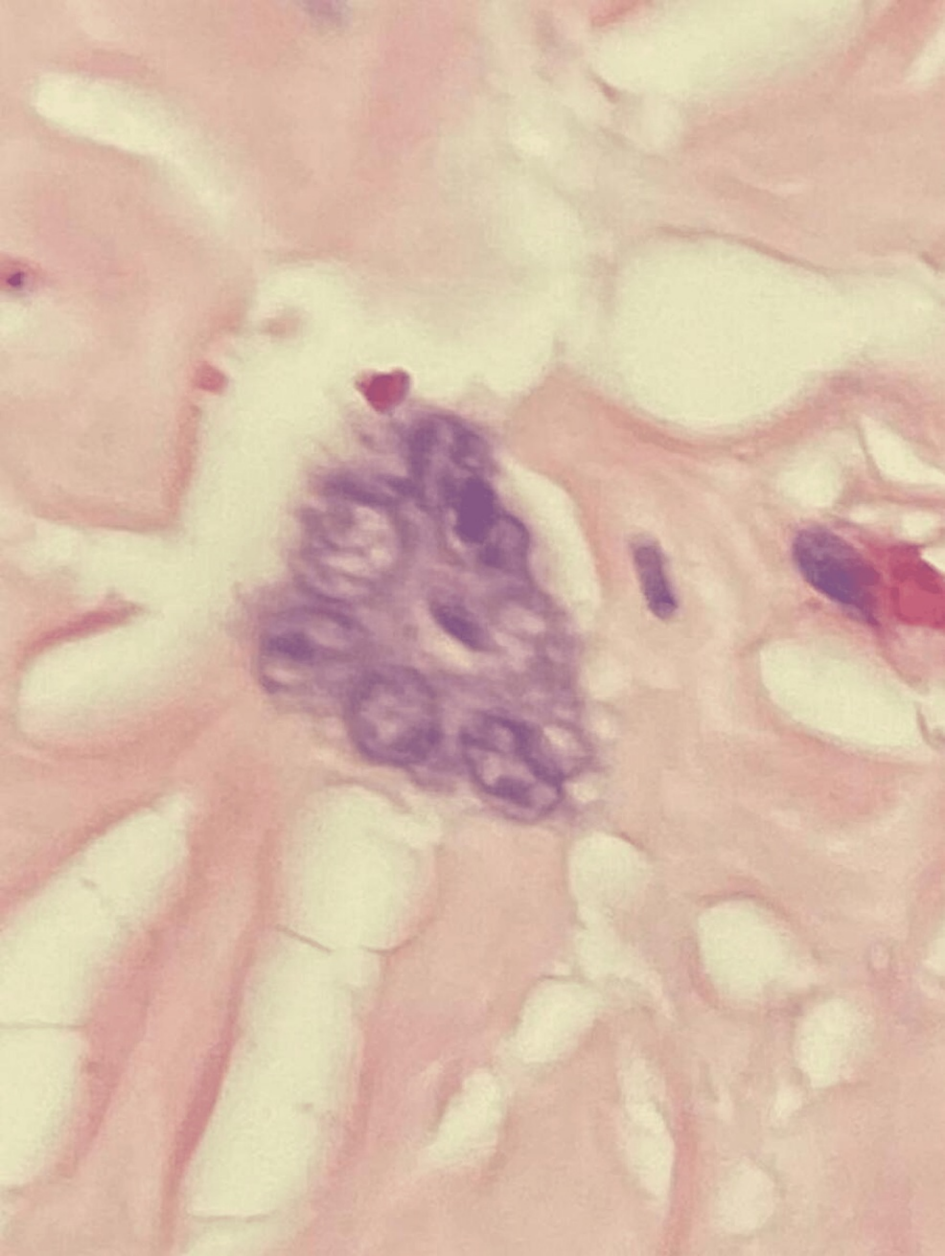
Multinucleated giant cell.

**Figure 3:**
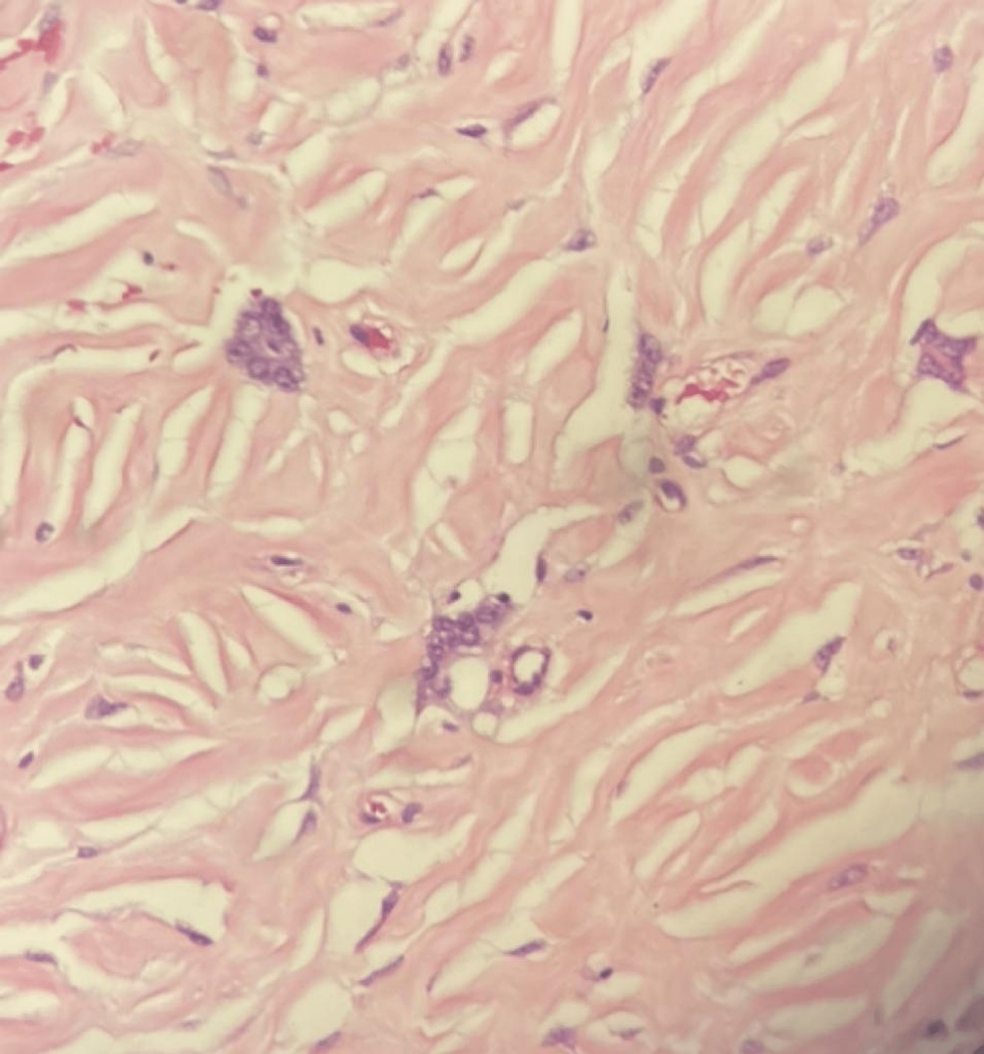
Stellate spindle-shaped and multinucleated cells. Mitotic figures are absent.

The clinical presentation was in favor of solitary neurofibroma versus atypical fibroxanthoma. However, an excisional biopsy revealed a polypoid lesion that showed the hypocellular proliferation of dermal spindle cells. The lesional cells were predominantly stellate spindle-shaped and multi-nucleated. Mitotic figures were absent (Figures 2-3). The overlying skin was thin and unremarkable. Immunohistochemical staining showed that the lesional multi-nucleated cells were negative for CD56, CD68, S-100, and factor XIlI. Given the presence of cellular atypia and the absence of necrosis or mitotic figures, a definitive diagnosis of PF of the skin was made based on the clinical presentation and immunohistochemical results. The patient was followed for five months and had no recurrent lesions.

## Discussion

PF is a rare benign fibrous neoplasm that typically manifests as a single asymptomatic lesion characterized by cellular atypia and pleomorphism without mitosis [3]. Similar atypia is seen in tumor cells of fibrohistiocytic skin tumors such as AFX, pleomorphic dermal sarcoma, and undifferentiated pleomorphic sarcoma. In contrast to the low cellularity and lack of mitosis in PF, these skin tumors often exhibit increased cellularity with a fascicular to storiform layout and extensive atypical mitosis with a high mitotic rate [4]. Recently, the World Health Organization distinguished AFX from pleomorphic dermal sarcoma given their indistinguishable clinicopathological features [5]. Both tumors commonly occur in the sun-exposed areas of the head and neck, followed by the upper trunk and extremities. However, AFX has low-grade malignant potential and never invades the subcutaneous fat, lymphovascular, or peri-neural areas-unlike pleomorphic dermal sarcoma. Moreover, AFX rarely metastasizes [1,2,4,6]. Similar to PF and AFX, pleomorphic dermal sarcoma cells react positively to CD68. However, this marker is not specific because it reacts positively to all types of fibrohistocytic skin tumors [2,4].

Neural tumors such as schwannoma and neurofibroma are other differential diagnoses of PF because they mimic the clinical and histopathological appearance of PF. Nevertheless, the absence of S-100 immunoreactivity in PF reliably distinguishes PF from these lesions. Furthermore, unlike schwannoma and PF tumors, neurofibroma is characterized by the presence of factor XIII. Accordingly, the absence of S-100 and factor XIII immunoreactivity in our case supported the diagnosis of PF among other differentials [7].

A final important differential is an atypical lipomatous tumor, also known as well-differentiated liposarcoma, that is categorized as an adipose tumor. This differential merits mention because some PF tumors contain adipose cells. In this situation, 12q15/MDM2 amplification by fluorescein in situ hybridization and MDM2 immunohistochemistry can pathogenetically distinguish PF from atypical lipomatous tumors. These markers are positive in atypical lipomatous tumors and negative in PF [6,8].

In a study of five patients with a strong personal or family history of confirmed pathogenic germline TP53 mutation, two out of the five patients had multiple PF lesions, suggesting that multiple PFs lesions in a young patient may be a sign of TP53-related hereditary cancer syndrome, which must be taken into consideration [9].

## Conclusions

We reported a case of a rare benign tumor known as PF of the skin. The lesion was surgically treated with simple excision and local recurrence was not observed for up to five months. The case highlights the importance of obtaining a full clinical picture, histopathology, and conducting immunohistochemistry to support an accurate diagnosis of PF.
